# QuEChERS and UPLC-MS/MS-Based Quantification of Human Plasma of Eight Nucleoside Reverse Transcriptase Inhibitors and Platinum Anticancer Drugs for Hepatocellular Carcinoma

**DOI:** 10.3390/molecules30102204

**Published:** 2025-05-18

**Authors:** Yanan Liu, Jiangning Peng, Yan Liang, Yilin Li, Xiaolan Zhen, Hui Li

**Affiliations:** 1 College of Chemistry and Pharmaceutical Engineering, Hebei University of Science and Technology, Shijiazhuang 050000, China; yananwenl@163.com (Y.L.); lyl10100909@163.com (Y.L.); 2 College of Pharmacy, Hebei Medical University, Shijiazhuang 050000, China; 16651498997@163.com (J.P.); liang_yan0523@163.com (Y.L.); 3 Hebei Institute of Drug and Medical Device Inspection, Shijiazhuang 050000, China; zhenxlan2007@163.com

**Keywords:** QuEChERS, UPLC-MS/MS, NRTIs, platinum, hepatocellular carcinoma, human plasma

## Abstract

Nucleoside reverse transcriptase inhibitors (NRTIs) and platinum-based chemotherapeutics are widely utilized in cancer treatment. Evidence suggests that drug plasma concentrations are closely linked to both therapeutic efficacy and the risk of adverse effects. Consequently, developing therapeutic drug monitoring (TDM) methods is essential. Here, an effective procedure utilizing QuEChERS (Quick, Easy, Cheap, Effective, Rugged, and Safe) techniques for preparing samples and UPLC-MS/MS for simultaneously measuring eight NRTIs and platinum-based drugs in human plasma is described. Chromatographic separation was conducted with an Agilent Eclipse Plus C18 column (4.6 × 100 mm, 3.5 μm) with acetonitrile with 0.1% formic acid as Phase A and 0.1% formic acid in water as Phase B, achieving complete separation within 10 min. The target analytes—lamivudine, telbivudine, emtricitabine, entecavir, tenofovir, nedaplatin, oxaliplatin, and adefovir dipivoxil—exhibited strong linearity within the 10–1000 ng/mL and 1–100 ng/mL ranges, showing correlations (r^2^) ≥ 0.9962. The method demonstrated excellent accuracy (−6.72% to 7.82%) and selectivity (84.53%–110.49%), as well as satisfactory recovery and stability. Overall, this analytical approach can be used to detect the combination of eight NRTIs and platinum-based drugs in human plasma. This method enables plasma drug-level monitoring in real time, with applications for individualized treatment approaches.

## 1. Introduction

Cancer represents a major health challenge, with a rising prevalence [1]. Hepatocellular carcinoma (HCC), the most common liver tumor, is linked to high levels of mortality, ranking fourth globally in cancer-related deaths [2]. Early detection and continuous monitoring of HCC improve the likelihood of curative treatment. However, even in regions with advanced healthcare systems, screening efforts remain insufficient.

Nucleoside reverse transcriptase inhibitors (NRTIs) are nucleoside and nucleotide structural analogs that competitively inhibit the binding of natural nucleosides to HIV-1 reverse transcriptase, thereby blocking viral DNA synthesis and suppressing viral replication [3]. Beyond their antiviral properties, NRTIs also exhibit inhibitory effects against chronic hepatitis B, reducing viral replication, alleviating liver damage, and ultimately exerting anti-tumor activity [4]. These agents are typically utilized for treating chronic hepatitis B, with drugs such as entecavir, lamivudine, telbivudine, tenofovir, emtricitabine, and adefovir dipivoxil playing a role in liver cancer treatment by inhibiting hepatitis B virus replication [5,6,7].

Platinum-based chemotherapeutics are cell cycle-nonspecific agents with broad-spectrum anti-tumor activity. These drugs interact with intracellular DNA, forming interstrand and intrastrand crosslinks or protein–DNA adducts, thereby disrupting DNA structure and function, ultimately inhibiting tumor growth. Platinum compounds are widely employed in the treatment of various solid malignancies, including HCC, as well as lung, esophageal, ovarian, gastric, breast, pancreatic, and lung cancers [8,9,10]. Compared to traditional cytotoxic chemotherapeutics, platinum drugs have the characteristics of strong anti-tumor activity and a wide anti-cancer spectrum. But they still have high systemic toxicity. For instance, nedaplatin, a second-generation platinum analog, exhibits enhanced water solubility, broad-spectrum anti-tumor efficacy, and reduced toxicity relative to first-generation platinum drugs [11,12]. Oxaliplatin, a third-generation platinum compound, exerts anti-tumor effects by binding to tumor cell DNA, thereby preventing replication and controlling tumor progression. It is commonly utilized for treating colorectal and liver cancers [13].

Despite their efficacy, these agents are associated with potential adverse effects, including hematologic toxicity, nephrotoxicity, nausea, vomiting, headache, and insomnia [14,15,16,17]. Therefore, maintaining optimal plasma drug concentrations is crucial to maximize therapeutic benefits while minimizing toxicity.

Given the clinical importance of these drugs, monitoring their plasma concentrations is essential for improving both efficacy and safety. Developing a rapid, reliable, and sensitive method for quantifying anti-HCC drugs is critical for optimizing clinical pharmacotherapy. HPLC-MS/MS is widely recognized for its use in quantifying plasma and whole-blood drug concentrations [18,19,20]. This technique offers advantages such as high selectivity, sensitivity, accuracy, small sample volume requirements, and rapid analytical throughput.

Drug concentration analysis typically involves various sample pretreatment techniques, including protein precipitation, liquid–liquid extraction, salt-assisted liquid–liquid extraction, and solid-phase extraction. The pretreatment of samples is critical for reducing matrix effects (MEs) and enhancing the purification process [21,22,23,24]. Among these approaches, QuEChERS (Quick, Easy, Cheap, Effective, Rugged, and Safe) was created for analyzing pesticide residues and has recently gained prominence in plasma drug and metabolite extraction [25].

Here, the development and validation of a rapid procedure for quantifying eight drugs simultaneously in human plasma is described. The UPLC-MS/MS method, combined with the QuEChERS sample preparation technique, enables the efficient, sensitive, and specific quantification of lamivudine, telbivudine, emtricitabine, entecavir, tenofovir, nedaplatin, oxaliplatin, and adefovir dipivoxil, offering a robust tool for therapeutic drug monitoring in clinical settings.

## 2. Results

### 2.1. Validation

The method was validated following the US Food and Drug Administration (FDA) Bioanalytical Method Validation Guidelines [26].

#### 2.1.1. Selectivity and Specificity

**Figure 1:**
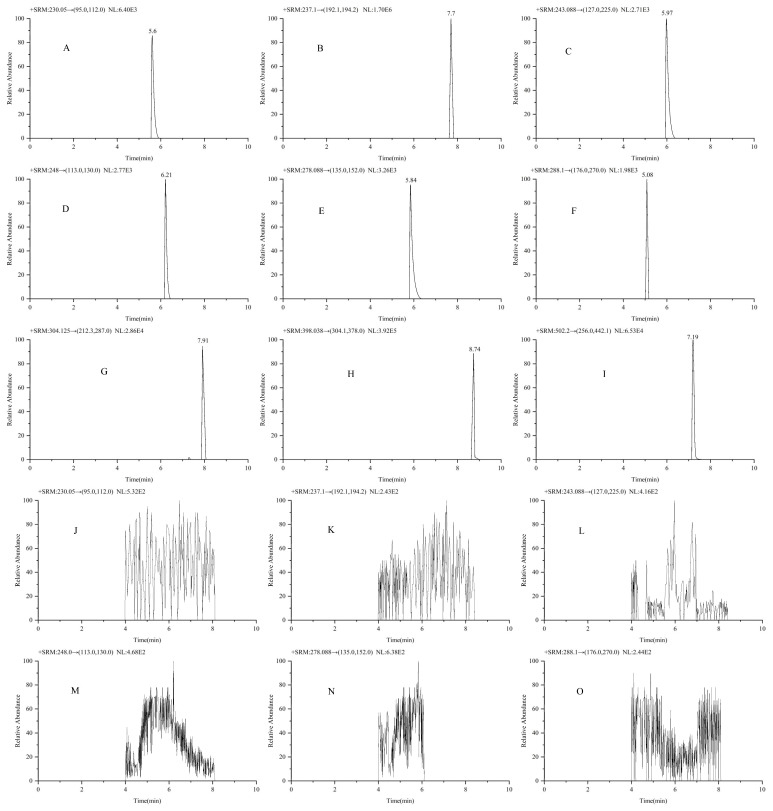

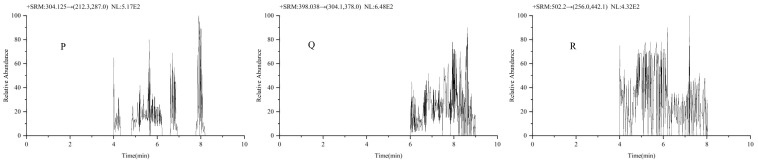
Typical chromatograms for lamivudine (**A**), IS—carbamazepine (**B**), telbivudine (**C**), emtricitabine (**D**), entecavir (**E**), tenofovir (**F**), nedaplatin (**G**), oxaliplatin (**H**), adefovir dipivoxil (**I**), and blank plasma plots (**J**–**R**).

To assess selectivity, analyses of six blank samples of plasma and six lower limit of quantification (LLOQ) samples were conducted, with these samples being from different individuals. Representative chromatograms of both sample types are illustrated in Figure 1. The retention times for lamivudine, telbivudine, emtricitabine, entecavir, tenofovir, nedaplatin, oxaliplatin, and adefovir dipivoxil, along with the internal standard (IS), were found to be 5.60, 5.97, 6.21, 5.84, 5.08, 7.91, 8.74, 7.19, and 7.70 min, respectively. The carryover of high standard concentrations to the blanks was below the proscribed limits of 20% of the LLOQ or 5% of the IS [27], indicating minimal residual effects and confirming the absence of analytical interference from any endogenous plasma components.

#### 2.1.2. Preparation of Standard Curves

Table 1 presents the linear equations, limits of detection (LODs), and limits of quantification (LOQs) for quantitative analysis. The calibration curves for entecavir, lamivudine, telbivudine, tenofovir, and emtricitabine (10–1000 ng/mL), as well as for adefovir dipivoxil, nedaplatin, and oxaliplatin, exhibited strong linearity. The sensitivity of the analytical method was evaluated using the correlation coefficient (R^2^), which was found to be ≥0.99 for all analytes. The LOD and LOQ were determined to be in the ranges of 0.089–4.236 ng/mL and 0.30–9.28 ng/mL, respectively.

#### 2.1.3. Precision and Accuracy

Precision and accuracy were assessed according to the FDA guidelines. Four concentrations of quality control (QC) samples were prepared within the range of calibration: LLOQ, low QC (LQC, 3 × LLOQ), medium QC (MQC, 30–50% of the calibration range), and high QC (HQC, ≥75% of the upper LOQ) [28]. Six replicates were utilized for assessments of accuracy and precision in the QC and LLOQ samples. The results, summarized in Table 2, demonstrated that intra-day accuracy ranged from −6.72% to 7.82%, with a relative standard deviation (RSD) ≤ 9.68%, confirming the method’s suitability for detecting the eight target drugs.

#### 2.1.4. Matrix Effects and Recovery

To evaluate ME and recovery, three QC samples differing in concentration were prepared, with each sample analyzed in six replicates. The results indicated a high efficiency of extraction for the eight drugs, with recovery rates between 84.53% and 110.49%. Matrix effects were found to be negligible, as demonstrated by an RSD ≤ 10.22% (Table 3).

#### 2.1.5. Stability

Methodological stability was assessed under different storage conditions by analyzing triplicate QC samples of both low and high concentrations (Table 4). The method demonstrated good stability, with an RSD ≤ 10.78% and QC sample recovery rates ranging from 88.77% to 110.33%. Furthermore, plasma samples stored at −20 °C for one month did not exhibit any significant degradation or signal loss. However, prepared samples should be analyzed promptly to prevent prolonged storage, which could affect the accuracy of experimental results.

## 3. Discussion

### 3.1. MS Conditions

**Figure 2:**
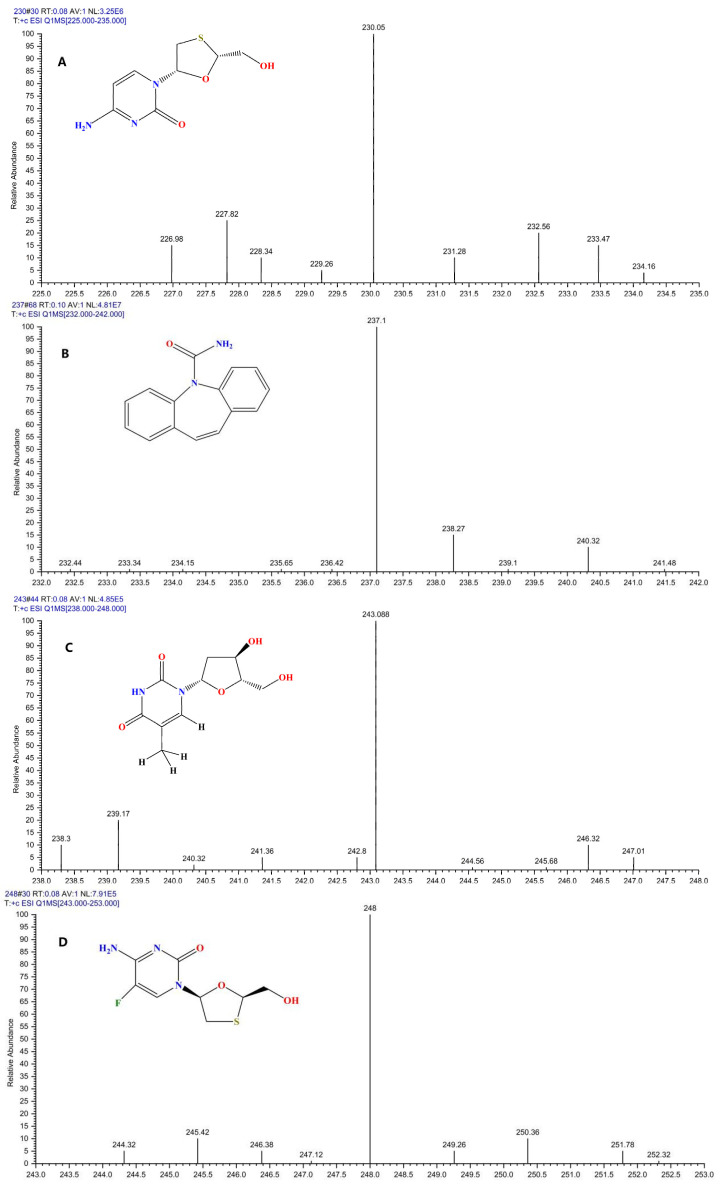

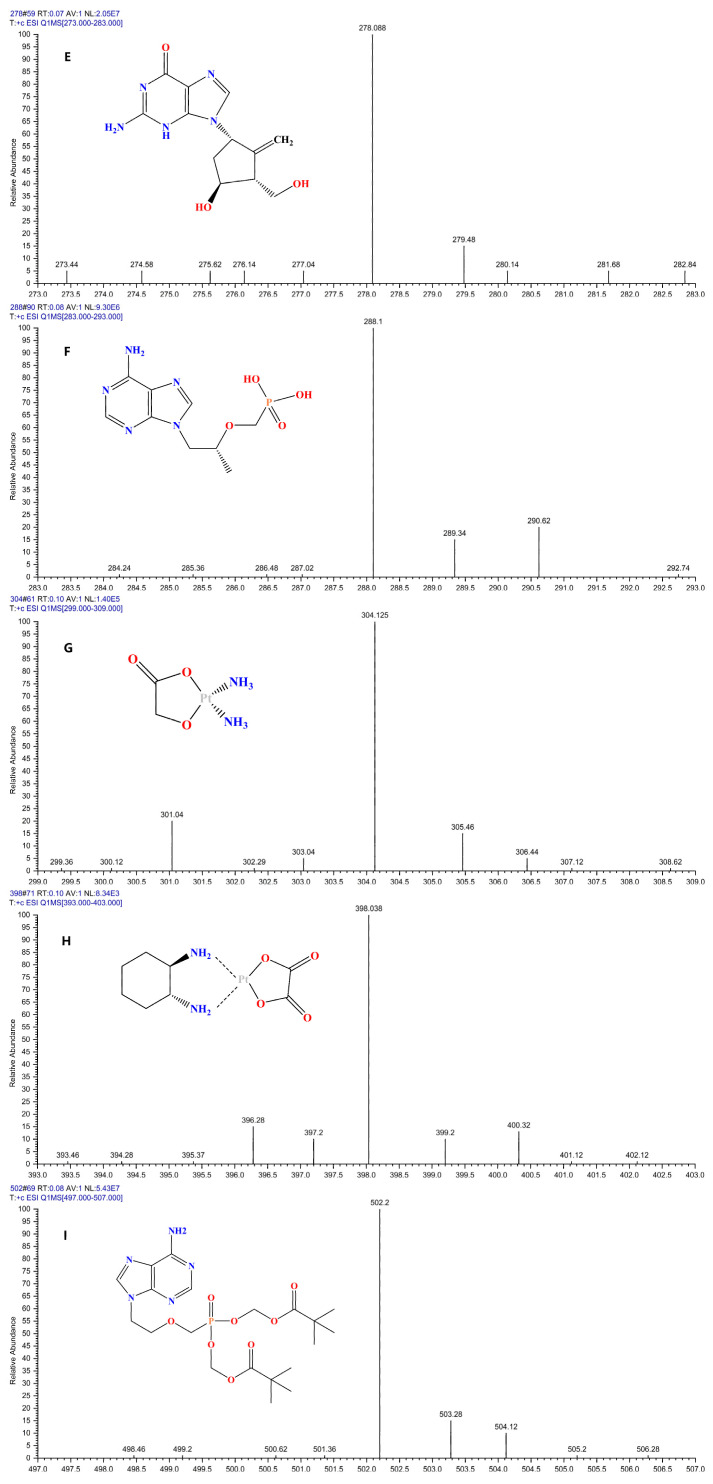
The product ion mass spectra in positive mode of lamivudine (**A**), carbamazepine (**B**), telbivudine (**C**), emtricitabine (**D**), entecavir (**E**), tenofovir (**F**), nedaplatin (**G**), oxaliplatin (**H**), adefovir dipivoxil (**I**), and their chemical structures.

MS optimization was achieved by directly injecting the target drugs using a syringe pump and performing scans in both positive and negative ion modes. These analyses revealed higher response intensities in positive ion mode for all analytes, leading to the selection of positive ionization for subsequent analysis. The parent ion spectra and structural formulas for each analyte and IS are presented in Figure 2.

### 3.2. MS Optimization

Various organic solvents were investigated for their effects on peak shape and response intensity. Acetonitrile provided a superior performance compared to other solvents. Additionally, the impact of different aqueous phases, including water, aqueous 0.1% formic acid, and aqueous 5 mmol/L ammonium acetate, was evaluated. The best peak shapes were obtained using 0.1% formic acid in water as the aqueous phase. Consequently, the mobile phase composition was finalized as 0.1% formic acid in water (Phase B) and acetonitrile with 0.1% formic acid (Phase A).

### 3.3. Pretreatment Condition Optimization

#### 3.3.1. Extractant Optimization

**Figure 3:**
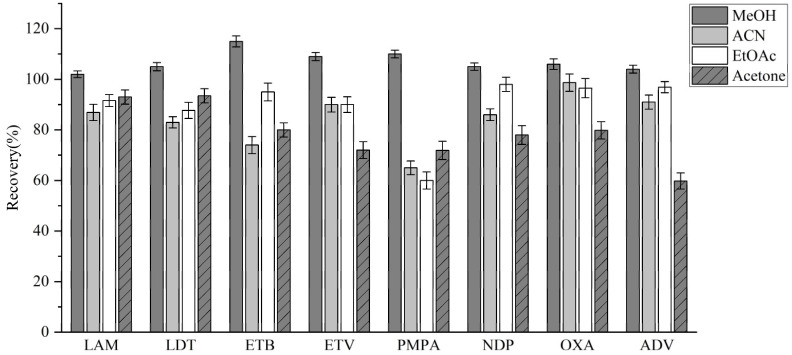
Recovery rates for different extractants.

The efficiency of commonly utilized organic solvents, including methanol, acetonitrile, ethyl acetate, and acetone, was evaluated based on analyte recovery rates. Equal 1 mL volumes of analyte solutions and each solvent were combined separately, and the extraction was performed in triplicate. The recovery rates of the eight tested drugs were then calculated. Among the solvents tested, ethyl acetate exhibited the lowest extraction efficiency, whereas methanol provided a superior analyte recovery and better peak shape compared to the other solvents (Figure 3). Consequently, methanol was chosen for extraction.

**Figure 4:**
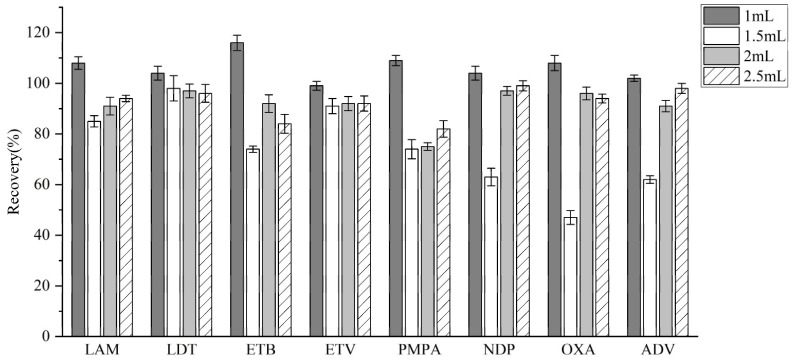
Recovery rates extracted with different volumes of methanol.

To further optimize the extraction process, different amounts of methanol (0.5, 1, 2, and 2.5 mL) were assessed for their impact on extraction efficiency. Additionally, various pretreatment techniques were evaluated to compare their effects on analyte recovery (Figure 4). Based on the results, the highest extraction efficiency was achieved with 1 mL of methanol, which was therefore selected.

#### 3.3.2. Selection of Salting-Out Agents

**Figure 5:**
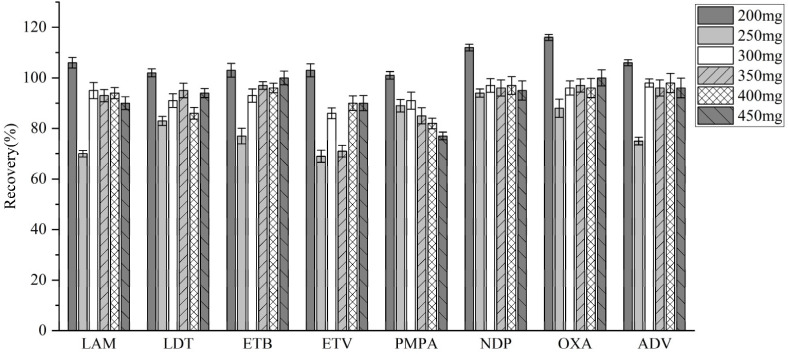
Recovery rates for analytes with different doses of MgSO_4_.

Anhydrous magnesium sulfate (MgSO_4_) was chosen to optimize the pretreatment of human plasma. To determine the optimal amount, different dosages ranging from 200 mg to 450 mg were tested. The effects of the various dosages on analyte recovery were then assessed (Figure 5). The results demonstrated that the highest recovery rate was achieved using 200 mg of anhydrous MgSO_4_. Therefore, this amount was determined to be optimal for further analysis.

#### 3.3.3. Adsorption Condition Optimization

**Figure 6:**
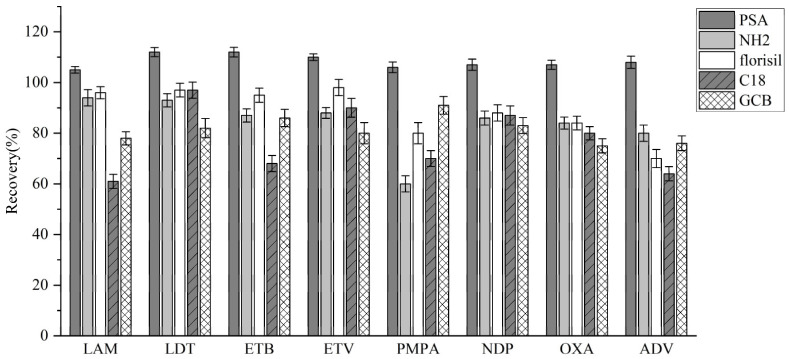
Recovery rates for different adsorbents.

Due to the complex composition of the plasma matrix, the addition of adsorbents was necessary to eliminate large molecules and potential interference. Five different adsorbents were tested, and recovery rates comparisons were made (Figure 6). PSA exhibited the most effective adsorption performance and was thus selected for further sample purification.

**Figure 7:**
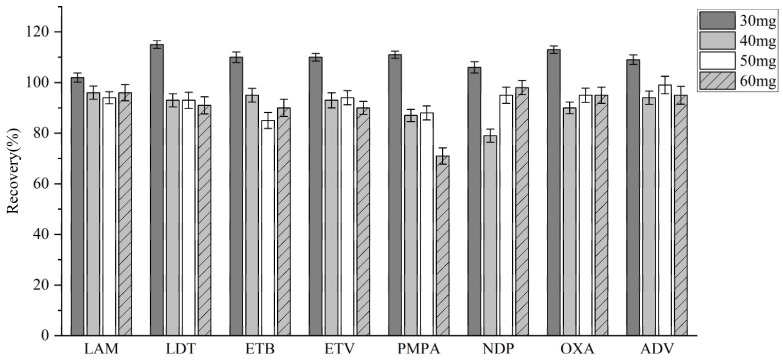
Recovery rates for PSA at different doses.

To determine the optimal PSA dosage, varying amounts (30, 40, 50, and 60 mg) were tested, and their effects on analyte recovery were evaluated (Figure 7). Of these dosages, 30 mg of PSA provided the best performance in QuEChERS pretreatment, making it the ideal choice for plasma sample purification.

In the previous work conducted by our research group, we applied QuEChERS pretreatment technology combined with LC-MS to evaluate the concentrations of multiple drugs. Compared with the determination of other similar drugs, the analysis of multiple mTOR inhibitors concentrations [28] and tyrosine kinase inhibitors (TKIs) concentrations in human plasma [29] showed good recovery rates. In addition, other members of our group also applied it to determine the concentration of drugs for treating hypertension and cardiovascular diseases. They successfully validated the practicality of this method as a means of detecting and quantifying target analytes in samples. Therefore, we believe that this QuEChERS technology is suitable for a wide range of drug monitoring fields.

## 4. Materials and Methods

### 4.1. Chemicals and Instruments

HPLC-grade methanol (MeOH), ethyl acetate (EtOAc), and acetone were purchased from Merck Drugs & Biotechnology (Darmstadt, Germany). HPLC-grade formic acid was purchased from Beijing Dikma Technology Co., Ltd. (Beijing, China); LC/MS-grade acetonitrile (ACN) was acquired from ThermoFisher Scientific Co., Ltd. (Shanghai, China). Analytical-grade anhydrous magnesium sulfate (MgSO_4_) was from Tianjin Damao Chemical Reagent Co., Ltd. (Tianjin, China). C18, Florisil, graphitized carbon black (GCB), NH_2_ and PSA were acquired from Agilent Technology Co., Ltd. (Beijing, China). The drugs lamivudine, telbivudine, emtricitabine, entecavir, tenofovir, nedaplatin, oxaliplatin, and adefovir dipivoxil were from Shanghai Yuanye Biotechnology Co., Ltd. (Shanghai, China).

A Vanquish Flex UHPLC and TSQ Altis Triple Quadrupole Mass Spectrometer (Thermo Fisher Scientific, Waltham, MA, USA) were used, as well as a nitrogen blowing concentrator (Beijing Pritech Instrument Co., Ltd., Beijing, China).

### 4.2. Chromatography

An Agilent Eclipse Plus C18 column (4.6 × 100 mm, 3.5 μm) was used with a mobile phase of acetonitrile with 0.1% formic acid (A) and an aqueous solution with 0.1% formic acid (B). The elution procedure was as follows: 100% B from 0 to 1 minute, decreasing to 80% from 1 to 1.5 min, further decreasing to 20% from 1.5 to 3 min, maintained at 20% from 3 to 5 min, increasing to 80% from 5 to 7 min, rising to 95% from 7 to 9 min, and returning to 100% from 9 to 10 min. The column temperature was 40 °C, with an overall run time of 20 min, an injection volume of 5 μL, and a flow rate of 0.420 mL/min.

### 4.3. MS

Analyses were conducted in positive ion mode using electrospray ionization (ESI) under selective reaction monitoring (SRM). The data analysis was conducted using Xcalibur software. The electric spray voltage was 3500 V, the sheath gas flow rate was 40 Arb, and the auxiliary gas flow rate was 10 Arb. The temperatures of the ion transfer tube and vaporizer were 275 °C and 350 °C, respectively. The collision gas was argon. Table 5 provides details of the monitored ions, collision voltages, and retention times for the individual analytes and the internal standard (IS).

### 4.4. Standard Preparation

To prepare the standard stock solution, 1 mg of each drug—lamivudine, telbivudine, emtricitabine, entecavir, tenofovir, nedaplatin, oxaliplatin, and adefovir dipivoxil—was dissolved in methanol to produce standard stock solutions of 0.1 mg/mL, which were kept at −20 °C.

For the mixed intermediate standard, 500 μL of the stock solutions of the individual standards were added to methanol to 1 μg/mL within a 50 mL volumetric flask. The solution was vortexed for 30 s and kept at −20 °C.

To prepare the working solution of mixed standards, appropriate stock solutions (0.1 mg/mL) were utilized to prepare calibration curve samples of 10 to 1000 ng/mL and 1 to 100 ng/mL. Specifically, the calibration levels for lamivudine, telbivudine, emtricitabine, entecavir, and tenofovir were 10, 25, 50, 100, 250, 500, 800, and 1000 ng/mL, while those for nedaplatin, oxaliplatin, and adefovir dipivoxil were 1, 2, 5, 10, 25, 50, 80, and 100 ng/mL. Carbamazepine (10 ng/mL) represented the internal standard.

### 4.5. Sample Pretreatment

Whole blood underwent centrifugation (3000× *g*, 20 min, 4 °C) and the plasma was aliquoted and kept at −80 °C. In total, 200 μL of plasma was added to 300 μL of the standards followed by vortexing for 30 s. Subsequently, 1 mL of methanol, 200 mg of anhydrous MgSO_4_, and 30 mg of PSA were introduced for salt precipitation and purification, and vortexed for 30 s, after which the sample was centrifuged (12,000× *g*, 10 min, 4 °C) and the supernatants dried under nitrogen and reconstituted in methanol (300 μL), followed by vortexing for 30 s, filtration through a 0.22 μm membrane, and injection.

### 4.6. Method Validation

We validated the selectivity, linearity, accuracy, precision, stability, and recovery rate of the method.

Selective analysis was conducted on 6 blank plasma samples from healthy individuals and 6 plasma samples containing internal standards and standard solutions by analyzing different sample evaluations. A response of interfering components below 20% of the analyte LLOQ and 5% of the internal standard is acceptable.

After preprocessing, the Xcalibur system was used to plot and perform linear regression analysis.

To evaluate accuracy, QC samples of different concentrations were analyzed, with each sample concentration analyzed three times. The intra-day precision was calculated by repeating the analysis of all sample concentrations three times a day, while the inter-day precision was calculated by repeating the analysis for six consecutive days.

We verified stability using QC samples of different prepared concentrations as follows: store samples at room temperature (25 °C) for 24 h, store in a sample tray (4 °C) for 24 h, freeze in the refrigerator (−20 °C) for 7 days, and evaluate long-term stability after 3 freeze–thaw cycles.

We calculated the extraction recovery rate by the ratio of the area of the QC sample to the area of the blank sample, added with the corresponding concentration standard solution after pretreatment.

### 4.7. Method Application

**Figure 8:**
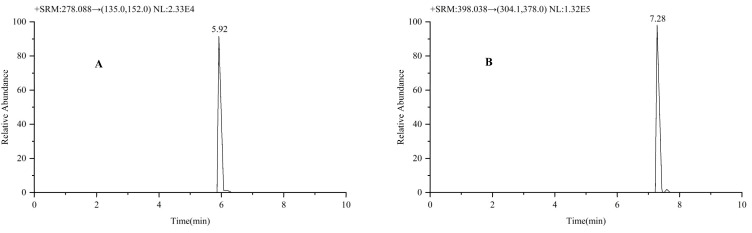
Patient chromatogram of entecavir (**A**) and oxaliplatin (**B**) combination therapy.

Due to the difficulties in obtaining blood from liver cancer patients, this approach is currently exclusively utilized to monitor entecavir and oxaliplatin blood concentrations. This approach has been tested and effectively used to quantify entecavir and oxaliplatin in the plasma of HCC patients. The fundamental information about the patient is as follows: a patient (69-year-old male) receiving entecavir dispersible pills once a day at a dose of 0.5 mg. We injected pirarubicin hydrochloride and oxaliplatin. Figure 8A,B show the chromatograms of entecavir and oxaliplatin combination therapy in the plasma of an HCC patient, respectively. The blood drug concentration was calculated to be 640 ng/mL. The results indicate that this method is suitable for detecting eight drugs in the plasma of liver cancer patients.

## 5. Conclusions

A novel technique for the simultaneous quantification of eight drugs in human plasma using UPLC-MS/MS was described. The procedure was optimized in terms of UPLC-MS/MS parameters and QuEChERS techniques to prepare samples for the simultaneous measurement of the levels of lamivudine, telbivudine, emtricitabine, entecavir, tenofovir, nedaplatin, oxaliplatin, and adefovir dipivoxil in human plasma. The method exhibited excellent accuracy, precision, and recovery while minimizing matrix effects. Additionally, the stability of the samples under various conditions was confirmed. Due to its simplicity, reliability, rapid execution, and high sensitivity, this method serves as a robust and effective tool for therapeutic drug monitoring.

## Figures and Tables

**Table 1:** Linear equations, LODs, and LOQs.

Analyte	Linear Equation	Linear Range (ng/mL)	R^2^	LODs (ng/ mL)	LOQs (ng/ mL)
Lamivudine	Y = 2.64 × 10^−4^X + 0.0478193	10–1000	0.998	2.281	7.60
Telbivudine	Y = 1.03 × 10^−4^X + 0.0780841	10–1000	0.999	2.344	7.81
Emtricitabine	Y = 5.68 × 10^−3^X + 0.00688369	10–1000	0.998	4.236	9.28
Entecavir	Y = 1.11 × 10^−4^X + 0.0259589	10–1000	0.996	2.381	7.94
Tenofovir	Y = 5.83 × 10^−3^X + 0.00739558	10–1000	0.9998	3.182	8.27
Nedaplatin	Y = 1.18 × 10^−3^X + 0.0182244	1–100	0.997	0.089	0.30
Oxaliplatin	Y = 7.67 × 10^−3^X + 0.0104116	1–100	0.9995	0.095	0.32
Adefovir dipivoxil	Y = 2.22 × 10^−3^X + 0.00575538	1–100	0.997	0.152	0.50

**Table 2:** Intra-day and inter-day precision analyses (n = 6).

Analyte	Nominal Concentration (ng/mL)	Intra-Day		Inter-Day	
		RE (%)	RSD (%)	RE (%)	RSD (%)
Lamivudine	10	−3.27	5.44	6.74	9.19
	25	3.52	1.87	2.48	1.02
	500	4.36	2.45	2.67	2.25
	800	−4.23	5.56	2.46	6.72
Telbivudine	10	2.18	6.42	−3.26	7.87
	25	4.62	7.88	5.74	8.66
	500	−2.95	1.45	−0.28	3.43
	800	−3.36	3.70	3.84	7.04
Emtricitabine	10	4.84	7.71	1.27	8.51
	25	−1.90	2.40	3.28	0.32
	500	6.30	3.03	−2.80	5.40
	800	4.27	1.91	1.43	6.02
Entecavir	10	5.07	8.43	3.06	7.52
	25	−2.46	1.25	1.21	4.40
	500	−2.84	1.95	2.94	1.85
	800	6.28	3.64	−0.94	6.05
Tenofovir	10	2.37	3.08	2.74	5.27
	25	5.78	6.22	3.81	5.83
	500	−3.21	2.35	5.27	6.05
	800	7.36	8.09	−4.06	6.39
Nedaplatin	1	7.82	9.35	1.68	4.97
	2	3.46	6.21	2.05	2.86
	50	−5.32	8.90	1.36	4.82
	80	3.26	7.34	−0.68	3.20
Oxaliplatin	1	−6.72	4.48	−2.47	9.68
	2	−3.46	7.25	1.38	4.04
	50	5.43	9.17	2.44	1.06
	80	5.89	6.68	5.42	4.90
Adefovir dipivoxil	1	−3.72	1.73	5.28	4.19
	2	4.58	5.61	3.81	7.01
	50	3.24	7.09	1.28	5.90
	80	1.36	2.52	2.34	1.89

**Table 3:** Recovery and matrix effect analyses (n = 6).

Analyte	Nominal Concentration (ng/mL)	Matrix Effect (%)		Recovery (%)	
		Mean	RSD	Mean	RSD
Lamivudine	25	93.45	3.84	101.65	7.49
	500	110.49	9.87	93.06	2.77
	800	87.02	2.16	108.94	1.49
Telbivudine	25	108.96	3.63	98.65	3.70
	500	97.78	2.76	93.32	1.29
	800	101.72	6.11	96.92	2.15
Emtricitabine	25	97.14	10.22	96.48	2.60
	500	109.73	4.14	96.25	3.76
	800	92.90	1.60	91.37	5.46
Entecavir	25	84.53	2.89	97.37	3.37
	500	89.25	4.84	96.54	1.09
	800	92.11	1.54	98.36	2.46
Tenofovir	25	104.19	1.31	106.57	2.03
	500	99.66	3.84	91.21	3.29
	800	102.40	7.96	101.26	3.45
Nedaplatin	2	97.70	1.48	90.41	8.03
	50	97.37	3.20	92.16	1.16
	80	105.42	3.11	95.25	2.75
Oxaliplatin	2	97.63	1.14	87.59	6.34
	50	105.31	3.45	91.12	1.55
	80	87.51	7.60	97.70	2.65
Adefovir dipivoxil	2	90.64	1.20	97.52	3.53
	50	98.61	4.54	97.01	6.57
	80	96.18	2.98	103.39	2.63

**Table 4:** Stability of QC samples under several different conditions (n = 3).

Analyte	Nominal Concentration (ng/mL)	Recovery% (RSD%)			
		25 °C/ 24 h	Autosampler (4 °C)/24 h	3 Freeze–Thaw Cycles	−20 °C/7 Days
Lamivudine	25	90.59 (2.21)	91.37 (7.98)	92.43 (9.94)	90.60 (1.38)
	800	94.04 (1.57)	98.95 (1.66)	96.78 (9.65)	95.89 (3.57)
Telbivudine	25	97.77 (6.74)	96.03 (1.99)	93.25 (3.81)	92.84 (3.31)
	800	110.33 (8.75)	98.49 (4.96)	89.84 (7.91)	93.45 (5.12)
Emtricitabine	25	107.86 (9.96)	104.94 (9.42)	102.32 (4.37)	96.26 (1.26)
	800	103.93 (2.15)	106.18 (2.44)	104.75 (3.89)	101.05 (1.35)
Entecavir	25	94.03 (1.47)	92.85 (1.58)	92.82 (9.28)	95.69 (3.98)
	800	94.96 (4.28)	94.66 (1.60)	97.45 (6.27)	97.35 (2.67)
Tenofovir	25	88.77 (2.25)	96.33 (1.64)	95.51 (1.32)	96.82 (6.78)
	800	89.23 (2.90)	93.84 (8.97)	101.96 (2.71)	92.56 (2.01)
Nedaplatin	2	94.18 (6.24)	98.19 (4.44)	94.58 (1.65)	98.40 (10.78)
	80	92.47 (6.35)	103.58 (2.57)	98.13 (1.71)	94.19 (4.69)
Oxaliplatin	2	95.29 (2.69)	93.83 (3.98)	96.89 (1.84)	90.81 (3.09)
	80	94.38 (5.58)	96.61 (2.12)	92.63 (1.26)	91.89 (1.21)
Adefovir dipivoxil	2	96.84 (1.03)	95.77 (8.90)	97.50 (2.59)	96.73 (9.02)
	80	98.12 (1.63)	98.60 (3.81)	95.31 (3.04)	91.34 (6.89)

**Table 5:** MS conditions for compounds and internal standards.

Compound	Precursor (*m*/*z*)	Product (*m*/*z*)	Collision Voltage (V)	RF Lens Voltage (V)	Retention Time (min)
Lamivudine	230.05	95.0 ^a^/112.0	13/38	30	0.77
Carbamazepine	237.1	192.1 ^a^/194.2	20/24	68	0.69
Telbivudine	243.088	127.0 ^a^/225.0	5/10	44	0.78
Emtricitabine	248	113.0 ^a^/130.0	12/38	30	0.78
Entecavir	278.088	135.0 ^a^/152.0	19/36	59	0.79
Tenofovir	288.1	176.0 ^a^/270.0	19/26	90	0.80
Nedaplatin	304.125	212.3 ^a^/287.0	12/22	61	0.73
Oxaliplatin	398.038	304.1 ^a^/378.0	5/27	71	0.77
Adefovir dipivoxil	502.2	256.0 ^a^/442.1	19/30	69	0.88

^a^ Quantification ion.

## Data Availability

Data are contained within the article.

## Abbreviations

The following abbreviations are used in this manuscript.

NRTIs	Nucleoside reverse transcriptase inhibitors
HCC	Hepatocellular carcinoma
LAM	Lamivudine
LDT	Telbivudine
ETB	Emtricitabine
ETV	Entecavir
PMPA	Tenofovir
NDP	Nedaplatin
OXA	Oxaliplatin
ADV	Adefovir dipivoxil
